# Untargeted metabolomics reveals differential metabolic pathways and biomarkers in the acute phase of Kawasaki disease

**DOI:** 10.3389/fmed.2026.1907194

**Published:** 2026-08-26

**Authors:** Hanqi Dai, Qianwen Wang, Yi Zhan

**Affiliations:** 1 Second Ward of Pediatrics, Jinhua Maternal and Child Health Hospital, Jinhua, Zhejiang Province, China; 2 Department of Pediatrics, Jinhua Municipal Central Hospital, Jinhua, Zhejiang Province, China; 3 Central Laboratory, Jinhua Municipal Central Hospital, Jinhua, Zhejiang Province, China

**Keywords:** biomarkers, differential metabolites, Kawasaki disease, plasma, untargeted metabolomics

## Abstract

**Introduction:**

Kawasaki disease (KD) is one of the most common rheumatic diseases in children and manifests with multisystem clinical features. Using untargeted metabolomics, our study investigated alterations in small-molecule metabolites in plasma of children with acute KD. Our study aimed to identify differential metabolic pathways and potential biomarkers.

**Methods:**

Plasma samples were collected from 30 children diagnosed with KD and 30 age-matched healthy controls (HC) at Jinhua Maternal and Child Health Hospital between January 2025 and December 2025. Liquid chromatography-tandem mass spectrometry (LC-MS/MS) was applied to analyse plasma samples. Enriched pathways were identified using the Kyoto Encyclopedia of Genes and Genomes (KEGG) pathway analysis, and differential metabolic pathways were determined using MetaboAnalyst 5.0. Differential metabolites were screened using the nonparametric Mann–Whitney *U*-test and receiver operating characteristic curve area (AUC). The conservative average AUC from nested cross-validation was reported as the primary performance metric. Pearson correlation analysis was conducted to evaluate correlations between metabolites and clinical parameters.

**Results:**

In total, 261 differential metabolites were identified between the KD and HC groups, including 87 lipids and lipid-like molecules, 69 organic heterocyclic compounds, 38 benzenoids, 34 organic acids, 14 phenylpropanoids, and 19 other compounds. Pathway analysis of these differential metabolites revealed 30 putatively enriched metabolic pathways for exploratory analysis. Of these pathways, primary bile acid biosynthesis, arginine biosynthesis, histidine metabolism, and phenylalanine-tyrosine-tryptophan biosynthesis were nominally associated with KD. Six metabolites with exploratory discriminatory performance (AUC > 0.8) were further identified: L-tyrosine, L-tryptophan, glutamine, histidine, histamine, and taurocholic acid. A combined model incorporating these metabolites achieved an apparent AUC of 0.984 in the full dataset; nested cross-validation yielded a more conservative AUC of 0.889 (95% CI 0.798–0.968), indicating promising exploratory discriminatory performance.

**Conclusion:**

Untargeted metabolomics enables identification of metabolically perturbed pathways during the acute phase of KD. L-tyrosine, L-tryptophan, glutamine, histidine, histamine, and taurocholic acid may serve as candidate biomarkers for acute phase of KD.

## 1 Introduction

Kawasaki disease (KD) is an acute febrile exanthematous disease primarily characterized by systemic medium and small vasculitis. It has become one of the most common rheumatic diseases and the leading cause of acquired heart disease in children (1–4). Diagnosis relies on comprehensive assessment of clinical manifestations including fever, conjunctivitis, mucosal lesions, and cervical lymphadenopathy. Fever is generally regarded as the primary indicator for evaluating treatment response. The absence of validated specific biomarkers, together with inadequate recognition of KD among primary care clinicians, frequently results in misdiagnosis and poses challenges to clinical management.

Metabolomics enables systematic analysis of dynamic alterations in small-molecule metabolites in organisms and can directly reflect disease progression (5). By identifying specific untargeted metabolic changes using liquid chromatography-tandem mass spectrometry (LC-MS/MS), correlating with clinical characteristics, and establishing diagnostic models. This has important clinical significance for the prediction of complications and the diagnosis of the acute phase of autoimmune diseases (6, 7). Metabolomic approaches have garnered extensive interest in research on KD progression and have uncovered metabolic dysregulations implicated in KD pathogenesis (8, 9). These metabolic abnormalities directly affect multiple metabolic pathways and directly or indirectly facilitate inflammatory and immune responses during KD development (10). Previous studies have reported elevated circulating levels of metabolites including citrate, succinate, linoleic acid, and arachidonic acid in patients with acute KD, with perturbations observed across multiple pathways such as energy metabolism, lipid metabolism, and tryptophan metabolism (8–12).

In this study, we performed untargeted LC-MS/MS analysis on plasma samples from children with KD. By integrating differential metabolites and their enriched metabolic pathways, we aimed to identify candidate metabolites biomarkers for KD, and conducted a correlation analysis between candidate metabolites and clinical parameters to explore the potential roles of plasma metabolites in the initiation and progression of KD.

## 2 Patients and study design

### 2.1 Participant recruitment

30 children diagnosed with KD at Jinhua Maternal and Child Health Hospital between January 2025 and December 2025 were enrolled. Another 30 healthy children during the same period served as healthy controls (HC). A total of 29 males and 31 females were recruited in the study. The study was approved by the Medical Ethics Committee of Jinhua Maternal and Child Health Hospital (2025-KY-022) and informed consent was obtained from the guardians of the participants. Inclusion criteria: (1) age 6 months to 12 years; (2) diagnosis of KD according to the Expert Consensus on Diagnosis and Acute-Phase Treatment of KD (2); (3) acute phase of the disease without prior treatment with intravenous immunoglobulin, corticosteroids, or aspirin; (4) no antipyretic use within 6 h before blood sampling and no antibiotic use within 24 h; (5) no special diet or probiotic use within the previous month. Exclusion criteria: (1) long-term use of medications or health supplements; (2) chronic diseases such as liver, kidney, or heart dysfunction, or inherited metabolic disorders; (3) history of chronic infections such as EBV, CMV, or tuberculosis. Plasma samples were collected during the acute phase (duration of fever ≤7 days).

### 2.2 Sample processing and collection

(1) 2 ml of peripheral blood was collected into EDTA-anticoagulated tubes. Samples were centrifuged at 3,000 rpm for 10 min at room temperature. The supernatant was collected and centrifuged again at 12,000 rpm for 10 min at 4℃. The resulting supernatant was aliquoted into 1.5 ml tubes and stored at −80℃. (2) Frozen plasma samples were thawed. One hundred µl of each sample was transferred to an EP tube and mixed with 300 µl of extraction solution. The mixture was vortexed for 30 s, ultrasonicated for 10 min, and left to stand at −40℃ for 1 h. Samples were then centrifuged at 12,000 rpm for 15 min at 4℃. The supernatant was transferred to sample vials for LC-MS/MS analysis. An equal volume of supernatant from all samples was pooled to prepare quality control (QC) samples, which were also analyzed.

### 2.3 Chromatography conditions

Chromatographic separation was performed using an ACQUITY UPLC HSS T3 column (100 mm × 2.1 mm, 1.8 µm, Waters). Mobile phase A consisted of 5 mmol/L ammonium acetate and 5 mmol/L acetic acid in water, and mobile phase B was acetonitrile. The gradient elution program was as follows: 0–0.8 min, 2%-70% B; 0.8–2.8 min, 70%-90% B; 2.8–5.3 min, 90%-99% B; 5.3–5.9 min, 99% B; 5.9–7.5 min, 2% B; 7.5–7.6 min, 2% B; 7.6–10.0 min, 2% B. The flow rate was 0.35 ml/min, the injection volume was 4 µl, and the column temperature was maintained at 40℃.

### 2.4 Mass spectrometry conditions

An electrospray ionization source was used, and data were acquired separately in positive and negative ion modes. The ion source temperature was 350℃. The capillary voltage was +3.8 kV in positive mode and −3.4 kV in negative mode. The sweep gas flow rate was 0 Arb, auxiliary gas flow rate was 15 Arb, and sheath gas flow rate was 50 Arb. Data acquisition was performed in full-scan and data-dependent acquisition modes. In each acquisition cycle, the full scan range was 70-1,050 Da, with a resolution of 70,000, an automatic gain control target of 3E6, and a maximum injection time of 100 ms. The top 5 precursor ions with intensity >100,000 were selected for MS/MS scanning. MS/MS resolution was 17,500, with a maximum IT of 50 ms. The dynamic exclusion time was set to 6 s.

### 2.5 Data processing and statistical analysis

For untargeted metabolomics analysis, the raw data is first processed using XCMS for peak detection, peak integration, and retention time alignment. Based on the aligned peaks, the XCMS package integrates the chromatographic peaks for each sample and extracts the first-order peak areas as quantitative information. Feature filtering based on QC samples: Low-quality ions with a missing rate exceeding 50% in QC samples or 80% in experimental samples are excluded. Missing values were then imputed using the K-Nearest Neighbors (KNN) method, and the imputed data were normalized by Probabilistic Quotient Normalization (PQN). Metabolite annotation followed the Metabolomics Standards Initiative (MSI) guidelines. All differentially abundant metabolites reported in this study are classified as MSI Level 2 (putative annotation). For metabolites detected by XCMS, a preliminary putative annotation was first performed by matching precursor m/z values against the HMDB and KEGG databases using the open-source software MetaboAnnotation. To further confirm metabolite identification, MS/MS fragment spectra were matched against an integrated multi-database spectral library compiled from HMDB, MS-DIAL, MassBank, and GNPS, with all entries uniformly cleaned and filtered to remove redundancy and errors. Matching was conducted using the MetaboAnnotation package in R, with the following acceptance criteria: (1) MS/MS similarity score (cosine similarity) ≥0.7; (2) precursor mass tolerance ≤20 ppm; and (3) at least 4 matching fragment ions. Only features meeting all three criteria were annotated as MSI Level 2. All automated matches were manually verified by two independent investigators to confirm fragmentation plausibility and to exclude isotopic peaks, adducts, and noise. Differential fold change analysis and partial least squares discriminant analysis (PLS-DA) were applied to calculate fold changes. Combined with Benjamini-Hochberg-adjusted *P*-values from Wilcoxon tests and variable importance in projection (VIP) scores from the PLS-DA model, differential metabolites were identified based on the following criteria: (1) fold change >1.2; (2) VIP >1; (3) *P* < 0.05. The non-parametric Mann–Whitney *U*-test was used to further screen differential metabolites between the two groups. Cluster analysis was performed using R version 4.4.2. Statistical analysis was performed using GraphPad Prism 8. Continuous variables were presented as mean ± standard deviation (x¯ ± s). Comparisons between two groups were performed using the Mann–Whitney *U*-test or independent-samples *t*-test. Categorical data were presented as frequency (*n*) and percentage (%), and between-group comparisons were performed using the chi-square test. Metabolic pathway enrichment analysis was conducted using MetaboAnalyst 5.0 (https://www.metaboanalyst.ca/). Receiver operating characteristic (ROC) curves were used to evaluate the diagnostic performance of the identified metabolites. To avoid feature-selection leakage and obtain a realistic estimate of model performance, a nested cross-validation procedure was used. In the outer loop, the full dataset (*n* = 60) was randomly partitioned into 10 folds. Within each outer training set, the entire feature-selection pipeline was repeated independently: first, differential metabolites were re-selected using the same criteria as in the primary analysis [(1) fold change >1.2; (2) VIP >1; (3) Benjamini-Hochberg adjusted *P* < 0.05]); second, univariate ROC screening (AUC > 0.8) was applied to these re-selected metabolites to identify candidate markers for the combined model. A logistic regression model was then trained on the metabolites selected within that training fold and evaluated on the corresponding outer validation set. This procedure was repeated for 200 iterations of 10-fold cross-validation, and the average AUC with 95% confidence interval (*CI*) was reported as the primary performance metric. The DeLong test was used to compare the AUCs between the combined model and individual metabolites. ROC curves were plotted from the predictive markers derived from the logistic regression analysis, and the corresponding the AUC, sensitivity and specificity with 95% *CI* were calculated. Pearson correlation analysis was performed using the “Hmisc” package in R to investigate the relationships between key metabolites and clinical characteristics. Heatmaps were created with the “ComplexHeatmap” package, and signiﬁcant correlations were visualized using scatter plots generated with the “ggplot2” package, offering insights into the metabolic alterations linked to clinical characteristics in the KD and HC group. A *P*-value <0.05 was considered statistically significant.

## 3 Results

### 3.1 Patients clinical parameters

A total of 60 children aged 6 months to 12 years were enrolled, mean age was (2.63 ± 1.82) years, body weight (13.12 ± 3.71) kg, and height (89.98 ± 15.47) cm. There were no statistically significant differences between the two groups in sex ratio, age, height, weight, or other baseline characteristics (*P* > 0.05). The levels of white blood cell count (WBC), neutrophil count (Neu), C-reactive protein (CRP), erythrocyte sedimentation rate (ESR), alanine aminotransferase (AST), total bile acids (TBA), interleukin-2 (IL-2), IL-5, IL-6, IL-8, IL-10 and interferon-γ were higher in the KD group, while albumin was lower in the KD group; these differences were statistically significant (*P* < 0.05). The clinical characteristics of patients are shown in Table 1.

**Table 1:** Comparison of clinical parameters between the KD and HC.

Clinical indicators	KD (*n* = 30)	HC (*n* = 30)	*t/*χ^2^	*P*
Gender (F/M)	16/14	15/15	*t* = −2.48	0.805
Age (year)	2.57 ± 1.79	2.69 ± 1.88	χ^2^ = 0.067	0.796
Weight (kg)	13.42 ± 3.59	12.82 ± 3.87	*t* = 0.617	0.54
Height (cm)	90.87 ± 15.31	89.10 ± 15.83	*t* = 0.44	0.661
WBC (×10^9^/L)	13.91 ± 5.46	11.85 ± 5.52	*t* = 2.94	0.005
Neu (×10^9^/L)	9.37 ± 4.12	7.10 ± 4.37	*t* = 2.07	0.043
CRP (mg/L)	68.97 ± 46.43	5.32 ± 5.36	*t* = 8.329	<0.001
ESR (mm/h)	64.57 ± 26.89	5.32 ± 5.36	*t* = 8.329	<0.001
TP (g/L)	67.67 ± 8.61	70.21 ± 2.98	*t* = −1.529	0.132
ALB (g/L)	38.84 ± 5.29	43.72 ± 1.90	*t* = −4.758	<0.001
GLB (g/L)	28.42 ± 8.31	26.68 ± 2.82	*t* = 1.09	0.28
TBIL (μmol/L)	11.17 ± 12.35	8.02 ± 2.77	*t* = 1.362	0.178
DBIL (μmol/L)	5.05 ± 2.73	5.48 ± 1.96	*t* = 1.984	0.45
IBIL (μmol/L)	5.06 ± 2.74	5.49 ± 1.93	*t* = −0.699	0.487
TBA (μmol/L)	63.1 ± 80.34	23.2 ± 10.58	*t* = 2.695	0.009
ALT (U/L)	48.57 ± 51.93	31.83 ± 19.22	*t* = 1.655	0.106
AST (U/L)	32.4 ± 69.44	6.58 ± 3.37	*t* = 2.034	0.047
IL-1β (pg/ml)	3.28 ± 7.27	3.02 ± 3.23	*t* = 1.285	0.26
IL-2 (pg/ml)	2.29 ± 1.44	1.44 ± 1.46	*t* = 2.556	0.025
IL-4 (pg/ml)	1.40 ± 1.26	1.40 ± 1.26	*t* = 1.778	0.081
IL-5 (pg/ml)	2.31 ± 2.00	1.26 ± 1.47	*t* = 2.31	0.024
IL-6 (pg/ml)	81.93 ± 72.07	16.54 ± 27.12	*t* = 4.65	<0.001
IL-8 (pg/ml)	37.57 ± 29.65	13.12 ± 12.14	*t* = 4.18	<0.001
IL-10 (pg/ml)	11.83 ± 12.56	34.83 ± 30.59	*t* = -3.74	<0.001
IL-12 (pg/ml)	2.05 ± 3.27	0.97 ± 1.14	*t* = 1.71	0.92
IL-17 (pg/ml)	4.44 ± 5.59	2.32 ± 2.44	*t* = 1.61	0.113
TNF-α (pg/ml)	2.32 ± 2.45	2.32 ± 2.45	*t* = 0.703	0.485
*γ*-IFN (pg/ml)	2.18 ± 2.39	0.95 ± 0.82	*t* = 2.664	0.01
α-IFN (pg/ml)	2.97 ± 23.60	3.66 ± 3.65	*t* = 1.134	0.37

HC, healthy controls; KD, Kawasaki disease; CAL, coronary artery lesions; F, female; M, male; WBC, white blood cell; Neu, neutrophil; CRP, C-reactive protein; ESR, erythrocyte sedimentation rate; TP, total protein; ALB, albumin; GLB, globulin; TBIL, total bilirubin; DBIL, direct bilirubin; IBIL, indirect bilirubin; TBA, total bile acids; ALT, alanine aminotransferase; IL, interleukin; TNF, tumor necrosis factor; IFN, interferon. *P* < 0.05 was considered statistically significant.

### 3.2 Analysis of total plasma metabolites

A total of 902 metabolites were putatively annotated in plasma using mass spectrometry libraries. These included 272 lipids and lipid-like molecules, 207 organic heterocyclic compounds, 149 benzenoids, 117 organic acids, 49 phenylpropanoids, and 108 other substances. Among these, the main lipid-like organic compounds included fatty acids, glycerophosphocholines, fatty acid esters, and bile acids. The main organic heterocyclic compounds included indoles, 1-benzopyrans, purines, and their derivatives. The main benzene-containing substances included diphenylmethane, benzoic acid, and their derivatives. The main organic acids included aryl sulfates, amino acids, peptides, and their analogues.

### 3.3 Multivariate statistical analysis of plasma metabolites

**Figure 1:**
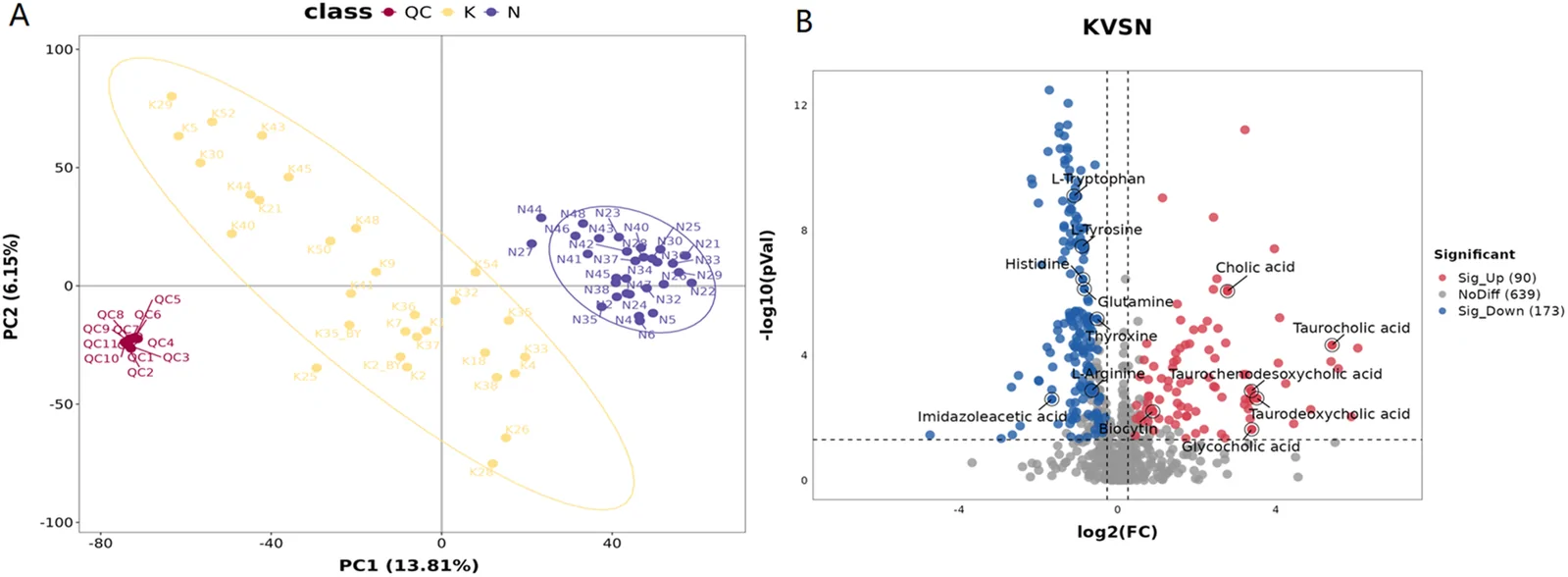
Partial least squares discriminant analysis (PLS-DA) model assessment. **(A)** PLS-DA score plot; **(B)** PLS-DA model validation. K, Kawasaki disease group; N, healthy control group.

PCA was employed to compare the proffles of KD, HC and QC group. Each point in the PCA plot represents a distinct sample (Figure 1A). The volcano diagram illustrates the changes in metabolites, showing that 90 metabolites were upregulated and 173 were downregulated. Each point represents a metabolite (Figure 1B).

**Figure 2:**
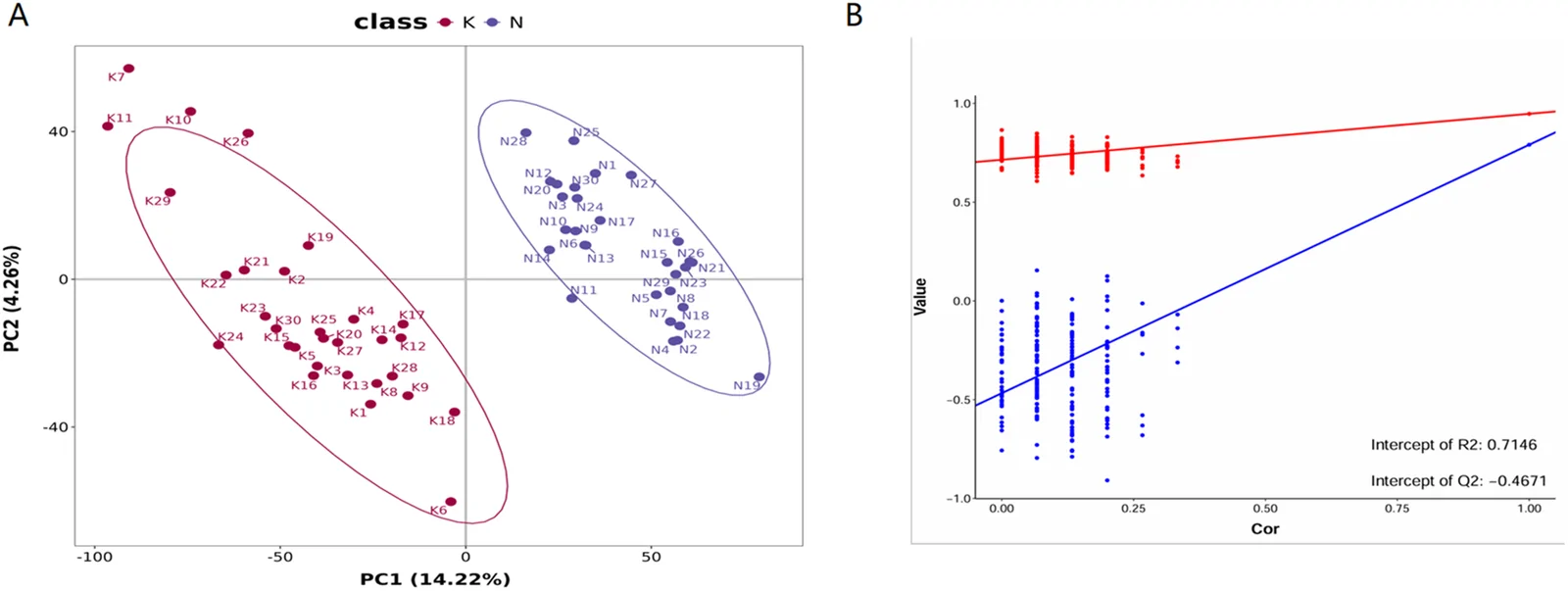
PCA and volcano diagram. **(A)** PCA showing the separation among the KD, HC and QC group based on their metabolic proﬁles; **(B)** The volcano diagram illustrates the upregulation and downregulation of metabolites. Red indicates upregulation, and blue indicates downregulation. QC, quality control group; K, Kawasaki disease group; N, healthy control group.

To accurately identify differential metabolites in plasma between the KD and HC groups, total peak area-normalized metabolic data from the two groups were analyzed using a PLS-DA model (Figure 2A). The PLS-DA score plot showed a clear separation between the two groups along the first PLS principal component. To avoid model overfitting, 7-fold cross-validation with 200 permutation tests was performed (Figure 2B). In the permutation test model, R^2^ = 0.7146, Q^2^ = −0.4671, indicating no overfitting and confirming the reliability of the PLS-DA model.

### 3.4 Screening of differential plasma metabolites between the two groups

The Mann–Whitney *U*-test was used to screen differential metabolites between the KD and HC groups. A total of 261 differential metabolites were identified, including 87 lipids and lipid-like molecules, 69 organic heterocyclic compounds, 38 benzenoids, 34 organic acids, 14 phenylpropanoids, and 19 other substances. The screening results of the top 20 differential metabolites are shown in Table 2.

**Table 2:** Screening results of the top 20 differentially metabolized substances in plasma between KD and HC.

Metabolite	RT (min)	m/z	FC	VIP	*P*
4-Methyl-6-tert-butylphenol	7.98	339.23	2.88	3.52	<0.001
Leucyl-glutamine	5.06	260.17	3.30	3.47	<0.001
IIILinalool oxide III	5.68	171.14	4.04	3.10	<0.001
3,4′,7-Trihydroxyflavone	4.44	271.06	1.25	2.90	<0.001
3,5-Di-tert-butyl-4-hydroxyphenyl	4.98	489.27	1.46	2.83	<0.001
Ouabain	3.65	585.29	3.30	2.79	<0.001
Apigenin 7-glucuronide	3.87	447.09	1.20	2.75	<0.001
Sucrose laurate	3.59	525.29	3.34	2.56	<0.001
Escitalopram	4.56	323.16	2.26	2.49	<0.001
N,N-Dimethylacetamide	0.70	88.08	2.21	2.44	<0.001
2 (3H)-Benzothiazolethione	4.96	165.98	2.03	2.39	<0.001
5-Methyl-2,5-di-1-pyrrolidinyl-2-cyclopenten-1-one	3.93	235.19	2.58	2.33	<0.001
Clomazone	3.73	238.08	2.50	2.33	<0.001
Taurocholic acid	4.41	533.33	5.49	2.29	<0.001
Phosphatidylcholine [17:2 (9Z,12Z)/0:0]	6.38	504.31	1.82	2.25	<0.001
Imidazoleacetic acid	1.13	149.02	2.83	2.24	<0.001
Tauro-α-muricholic acid	4.41	516.30	5.47	2.22	<0.001
Hydroquinone sulfate	2.67	188.99	1.75	2.22	<0.001
2-Ethyl-2-oxazoline	1.95	100.08	2.00	2.15	<0.001
4-Vinylphenol sulfate	3.88	199.01	1.50	1.93	<0.001

HC, healthy controls; KD, Kawasaki disease; RT, retention time; m/z, mass-to-charge ratio; FC, fold change; VIP, variable importance in projection.

### 3.5 Changes in differential plasma metabolites between groups

**Figure 3:**
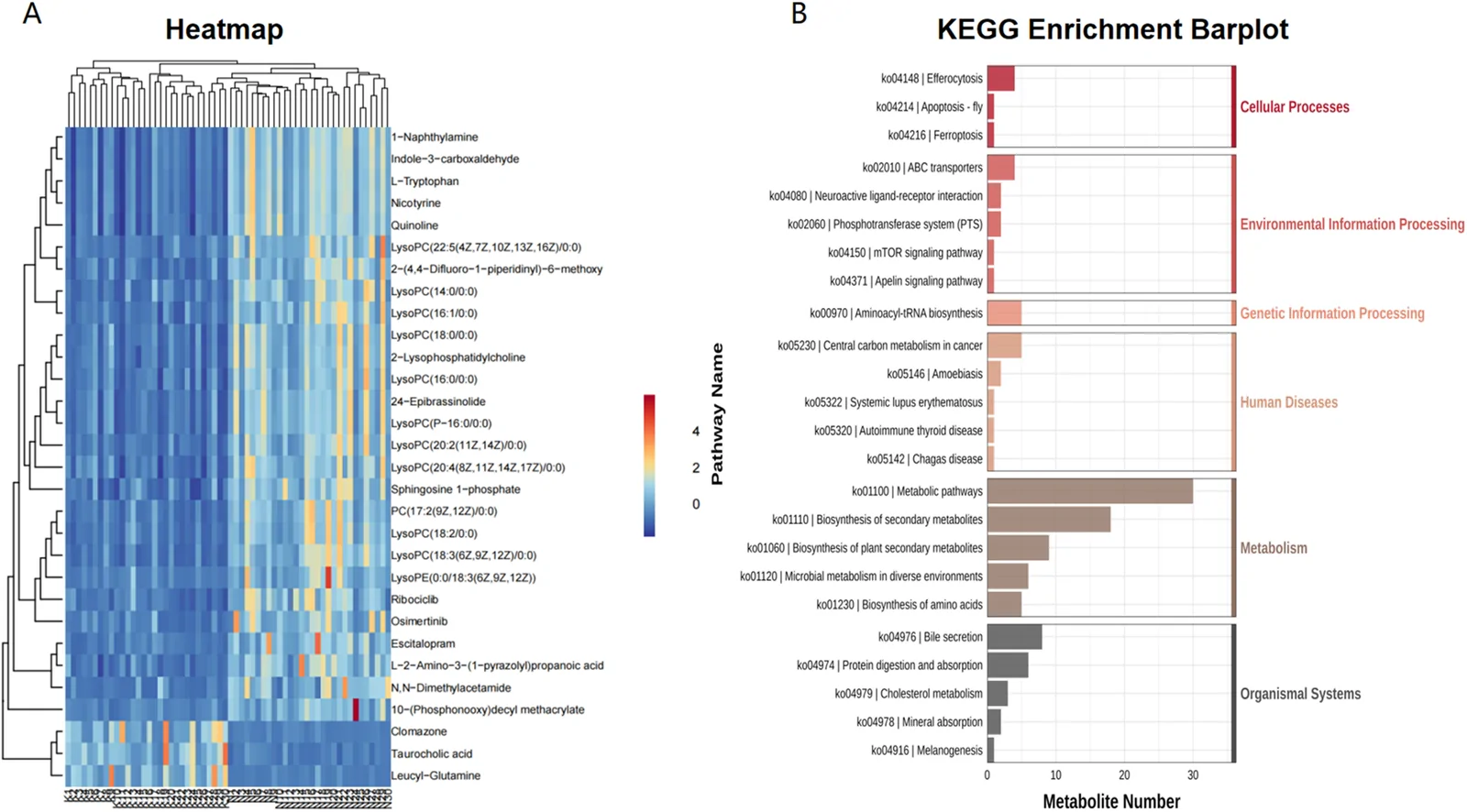
Analysis of differential metabolites and metabolic pathways in acute Kawasaki disease. **(A)** Clustered heatmap of the top 30 differential metabolites between acute KD and controls; **(B)** Enrichment analysis of dysregulated metabolic pathways in acute KD.

Heatmap and cluster analysis of the relative abundance changes of differential metabolites between the KD and HC groups are shown in Figure 3A. Differential metabolites in the KD group showed that bile acids (taurocholic acid) and peptides (leucyl-glutamine) were relatively more abundant than HC group. In contrast, amino acids (nicotyrine) and indole-3-carboxaldehyde were relatively more abundant than in the KD group. KEGG pathway enrichment identified bile acid secretion and metabolism, and the mTOR signaling pathway (Figure 3B).

### 3.6 Pathway analysis of differential plasma metabolites in KD

**Figure 4:**
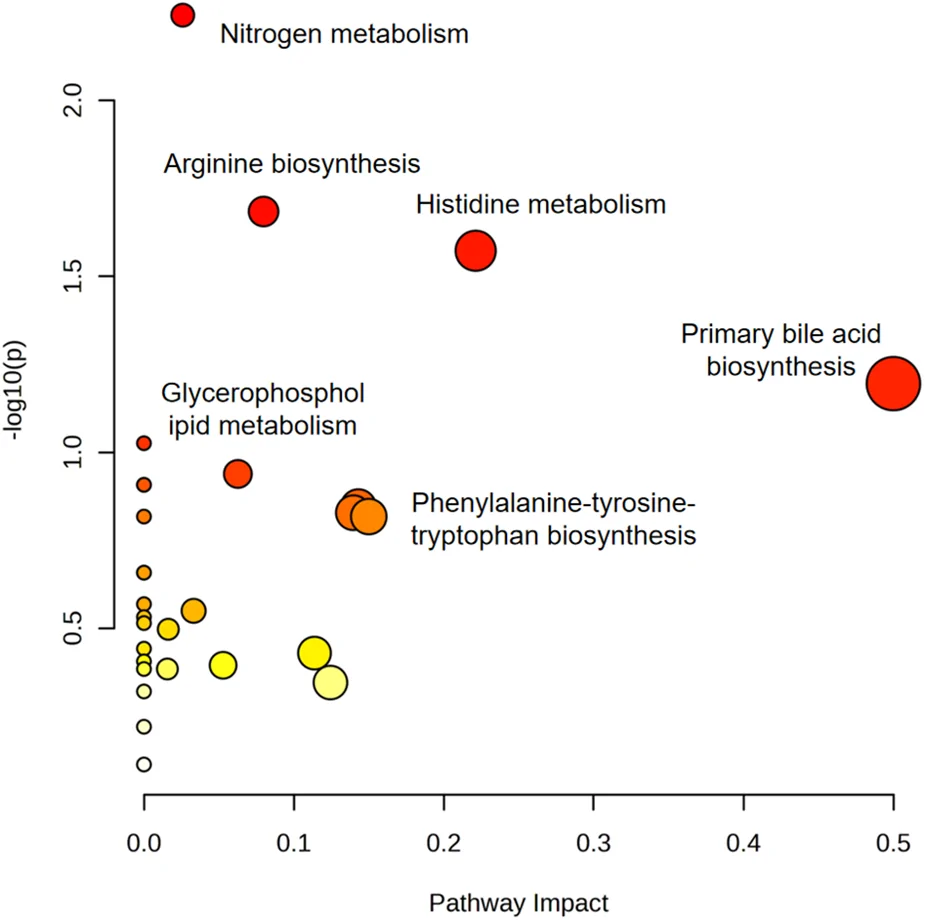
Enriched metabolic pathways of characteristic differential metabolites in Kawasaki disease. The *x*-axis represents the pathway impact, and the *y*-axis represents the -log10 of the *P*-value.

**Figure 5:**
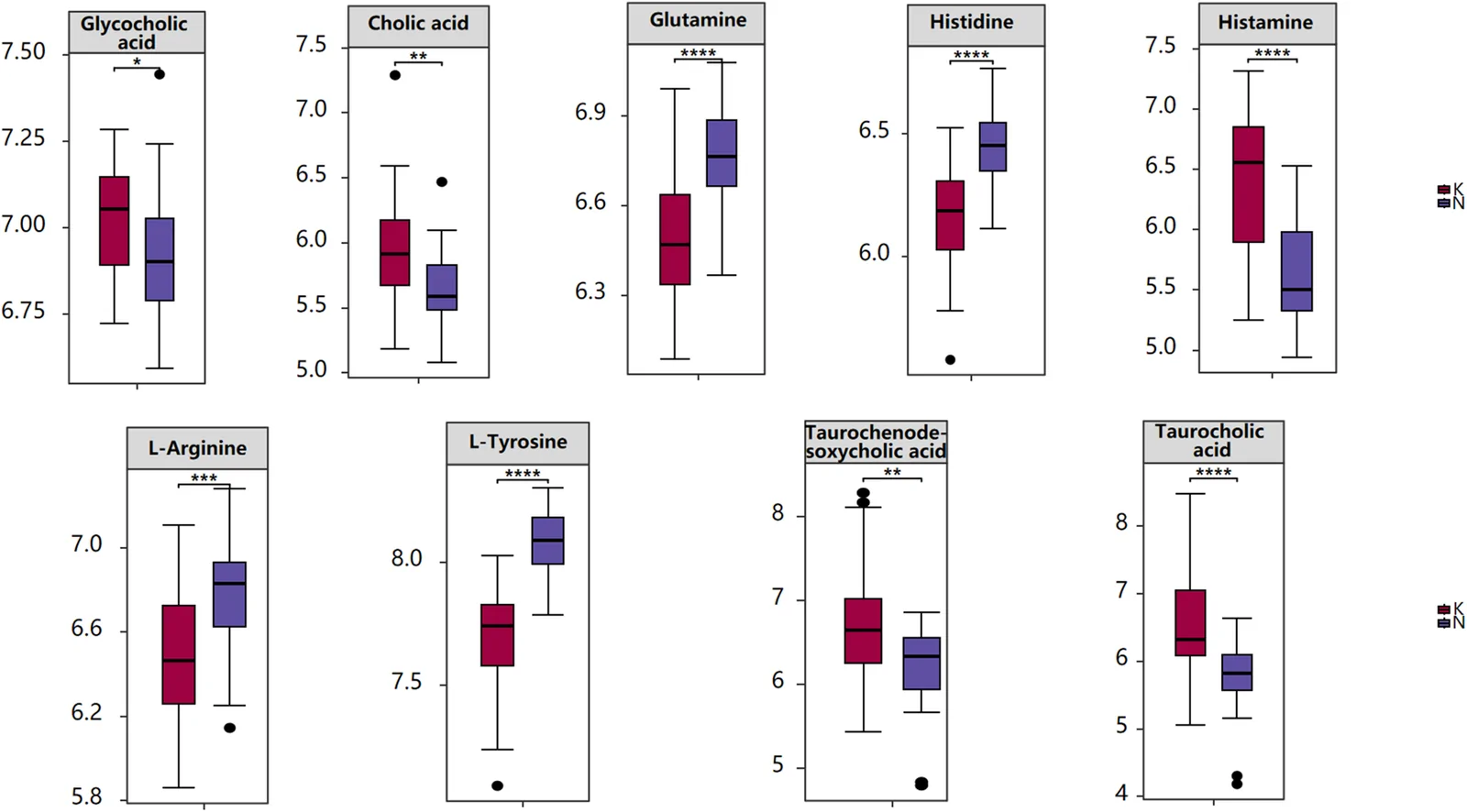
Expression differences of 9 differential metabolites between acute Kawasaki disease and healthy controls. K, Kawasaki disease group; N, healthy control group.

To further explore differences in metabolic pathways during the acute phase of KD, metabolic pathway analysis of the differential metabolites was performed using MetaboAnalyst 5.0 (Figure 4). A total of 30 metabolic pathways were annotated. The nominally enriched pathways associated with these differential metabolites included arginine biosynthesis, histidine metabolism, phenylalanine-tyrosine-tryptophan biosynthesis, and primary bile acid biosynthesis (Table 3). Comparisons of the major pathways identified 9 differential metabolites. In primary bile acid biosynthesis, an important pathway for substance metabolism, glycocholic acid, cholic acid, taurocholic acid, and taurochenodeoxycholic acid were all significantly elevated. In the arginine biosynthesis pathway, L-arginine and glutamine were significantly reduced. In the histidine metabolism pathway, histidine was significantly reduced, while histamine was significantly elevated. Within the phenylalanine-tyrosine-tryptophan biosynthesis pathway, L-tyrosine exhibited significantly reduced (Figure 5).

**Table 3:** Nominally enriched differential metabolic pathways in the acute phase of KD.

Pathway name	Match status	ER	*P*-value	FDR	Impact	Match metabolites
Phenylalanine-tyrosine-tryptophan biosynthesis	1	15.3076	0.0638	1	0.15	L-tyrosine
Histidine metabolism	2	7.6537	0.0268	0.7133	0.2213	histidine, histamine
Arginine biosynthesis	2	8.7474	0.0207	0.7133	0.0798	L-Arginine, glutamine
Primary bile acid biosynthesis	4	5.3244	0.0057	0.4582	0.5	glycocholic acid, taurocholic acid, taurochenodeoxycholic acid, cholic acid

ER, enrichment ratio; FDR, multiple testing-adjusted pathway *P*-values; Impact, impact value; KD, Kawasaki disease.

### 3.7 ROC curve analysis of combining metabolites

**Figure 6:**
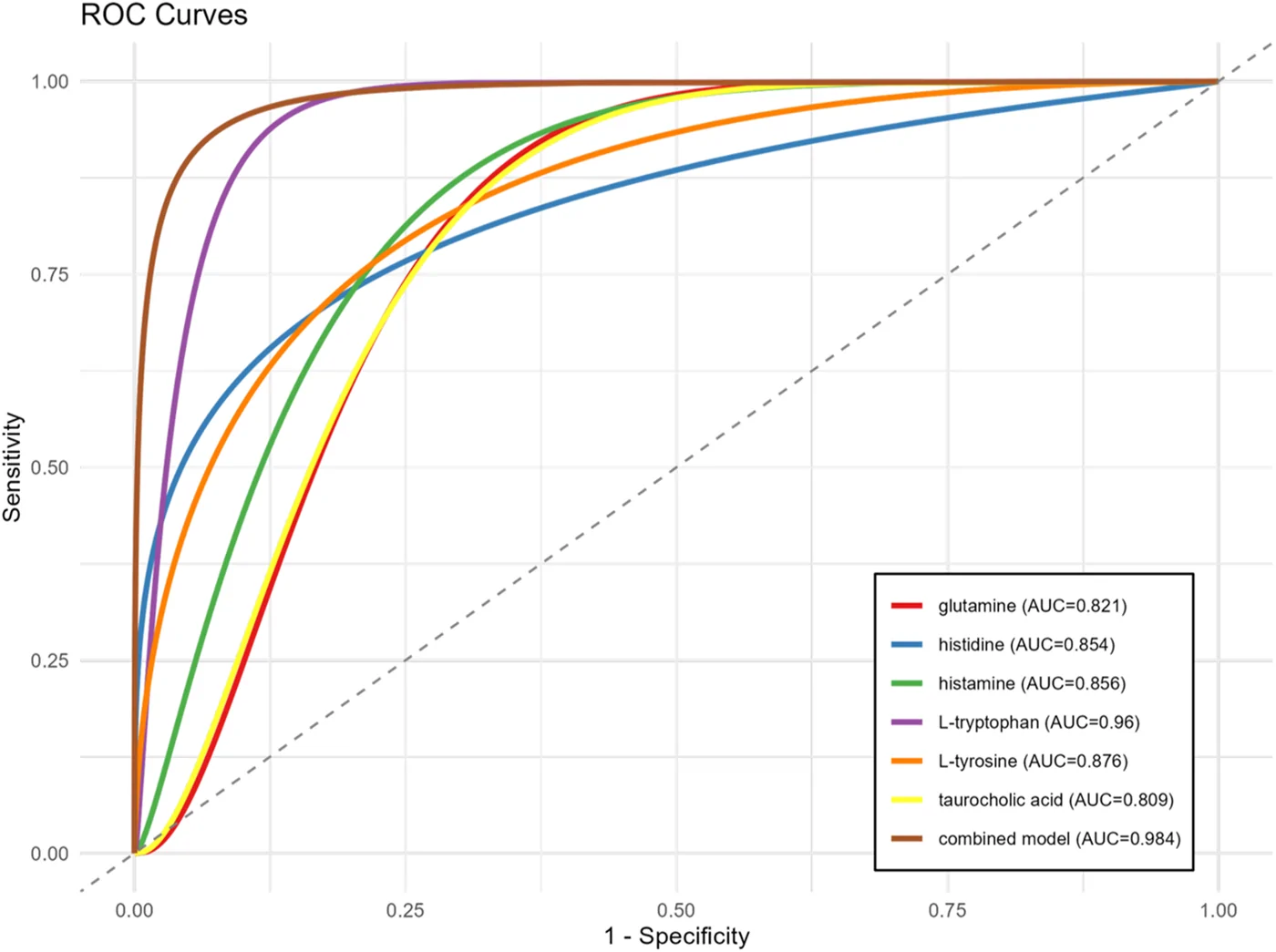
ROC curves demonstrating the discriminatory performance of a panel of metabolites.

Based on the above differential metabolites, ROC analysis was performed to identify candidate metabolites for KD using an AUC > 0.8. Six statistically significant metabolites were further identified as: glutamine, histidine, histamine, L-tryptophan, L-tyrosine, and taurocholic acid. The specific data was presented in Table 4. The logistic regression retained the six aforementioned metabolites in combined model. The unadjusted AUC of this combined model in the full dataset was 0.984 (Figure 6). However, to provide a more realistic estimate of its generalizability, we applied a 200-times repeated 7-fold cross-validation. The bias-corrected mean AUC was 0.921 (95% *CI*: 0.879–0.957), confirming that the model possesses excellent discriminative ability with a modest optimism of 0.063. DeLong test analysis further demonstrated that the cross-validated AUC of the combined model was significantly superior to that of any single metabolite (all *p* < 0.05). To rigorously account for the optimism potentially introduced by the prior feature-selection step, we performed an additional nested cross-validation in which the feature-selection procedure was repeated within each training fold. The pooled AUC across all outer test folds was 0.889 (95% Bootstrap *CI*: 0.798–0.968) (Supplementary Table S1; Supplementary Figure S1).

**Table 4:** Discriminatory performance in distinguishing patients with KD from the HC.

Metabolites	Sensitivity	Specificity	*P*-value	AUC	Asymptotic 95% *CI*	
					Lower bound	Upper bound
glutamine	0.9	0.7	<0.001	0.821	0.719	0.934
histidine	0.767	0.867	<0.001	0.854	0.774	0.955
histamine	0.7	0.867	<0.001	0.856	0.748	0.949
L-tryptophan	0.933	0.933	<0.001	0.96	0.93	1
L-tyrosine	0.963	0.7	<0.001	0.876	0.829	0.976
taurocholic acid	0.733	0.833	<0.001	0.809	0.712	0.926
combined model	0.967	0.933	<0.001	0.984	0.984	1

HC, healthy controls; KD, Kawasaki disease; ROC, receiver operating characteristic; AUC, area under the curve; *CI*, confidence interval; *P*-value <0.05 was considered statistically significant.

### 3.8 Correlation analysis between the biomarkers and clinical characteristics

A correlation heatmap was used to visually assess the associations between the six differential metabolites and 12 clinical parameters across the two study groups. Positive correlations are shown in red, whereas negative correlations are shown in blue. Colour intensity and the number of asterisks denote the magnitude of the correlation coefficient.

**Figure 7:**
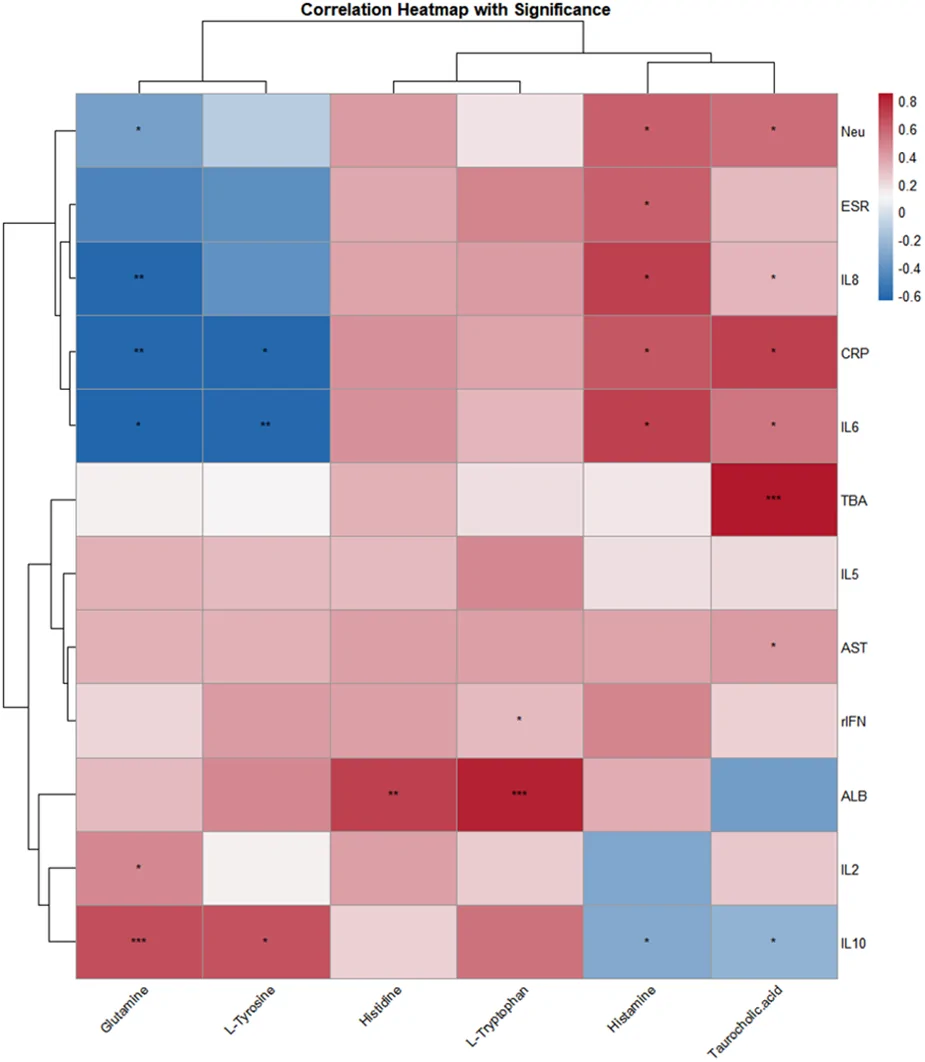
Heatmap analyses of metabolites and correlations with clinical indicators.

In KD patients during acute phase, taurocholic acid showed positive correlation with Neu, serum IL-6, IL8, TBA, AST, CRP and was negative correlated with serum IL-10. Histamine exhibited positive correlation with ESR, Neu, serum IL-6, IL8, CRP and was negative correlated with serum IL-10. L-tyrosine and glutamine were negative correlated with serum IL-6 and CRP, and positively correlated with serum IL-10. In addition, L-tryptophan and histidine were positively correlated with ALB (Figure 7).

## 4 Discussion

Kawasaki disease (KD) is a multisystem disorder characterized by fever and changes in the mucous membranes, skin, and lymph nodes (1). The coronary artery damage it causes has become the leading form of acquired heart disease in children (1–3). Up to 25% of children with KD who do not receive intravenous immunoglobulin eventually develop coronary artery lesions (CAL), although most cases are clinically silent and may only be discovered years later upon sudden death or myocardial infarction (3). Current clinical diagnosis relies on a combination of characteristic features and non-specific laboratory tests. However, individual variability in clinical presentation and the marked changes in inflammatory factors at different disease stages limit the diagnostic utility of these variables. In recent years, multi-omics approaches have provided new insights into the pathogenesis and diagnosis of KD.

Compared with genomics, metabolomics provides a more direct readout of the current biological state of an organism, exhibits tighter correlation with transcriptomic phenotypes derived from differentially expressed genes, and enables direct detection of terminal metabolites driving disease initiation (5). Few studies have used metabolomics approaches to characterize plasma metabolite alterations in children with acute-phase KD; existing studies are primarily small-sample studies with limited depth in the analysis of metabolic pathways. Zhang Jun et al. recruited 22 children with KD and found that sphingolipid metabolism, caffeine metabolism, and tyrosine metabolism pathways were differentially expressed between children with KD and healthy children. They proposed sphingosine, dihydronerveamide, phytosphingosine, and 3-dehydro-dihydrosphingosine as potential biomarkers for KD (9). Multiple studies have demonstrated elevated circulating levels of diverse metabolites including citrate, succinate, linoleic acid, and arachidonic acid in acute KD (8–10). The relationship between arachidonic acid metabolism and the pathogenesis of KD is relatively well established; arachidonic acid metabolites act as inflammatory mediators involved in the pathogenesis of KD, and the inhibitory activity of the arachidonic acid pathway acts as a clinical marker for evaluating aspirin efficacy (13). Arachidonic acid is converted to prostaglandin H2 via cyclooxygenase-2 (COX-2); COX-2 gene polymorphisms among Chinese population are associated with KD susceptibilityand modulate arachidonic acid fluctuations during the acute phase of KD (14). Furthermore, aspirin exerts antiplatelet effects mainly by irreversibly inhibiting cyclooxygenase-1 (COX-1) to block arachidonic acid-mediated platelet aggregation. Therefore, platelet inhibition measured via the arachidonic acid pathway can directly reflect antiplatelet efficacy. We hypothesized that antipyretic therapy administered during acute KD may interfere with arachidonic acid-related metabolites to a certain extent. Recent evidence indicates that studies exploring the associations between arachidonic acid-associated metabolites and proinflammatory cytokines in acute KD remain scarce. In our study, we performed untargeted metabolomics analysis using liquid chromatography–tandem mass spectrometry (LC-MS/MS) to profile plasma metabolite changes in children with acute-phase KD. We identified 261 altered metabolites. Combining metabolic pathway enrichment analysis and ROC analysis, we exploratorily identified that arginine biosynthesis, histidine metabolism, phenylalanine–tyrosine–tryptophan biosynthesis, and primary bile acid biosynthesis represent pathways linked to systemic vasculitic inflammation in KD. Furthermore, glutamine, histidine, histamine, L-tryptophan, L-tyrosine, and taurocholic acid (TCA) were correlated with levels of proinflammatory and anti-inflammatory factors, suggesting their involvement in immune imbalance during the acute phase of KD.

Primary bile acid biosynthesis refers to the biological process by which bile acids are synthesized from cholesterol. Besides participating in the enterohepatic circulation of bile acids, the metabolites also influence immune cells in the lamina propria of the intestine, regulate intestinal immune responses and microbial composition, and consequently affect systemic immune responses (15). Systemic vascular inflammation caused by KD can involve hepatic interlobular arteries and biliary endothelial cells, potentially resulting in gallbladder perfusion abnormalities. In addition, inflammatory cytokines have been reported to inhibit the activities of hepatic lipase and lipoprotein lipase, directly affecting gallbladder metabolic function and promoting the toxic accumulation of bile acid metabolites, which may persist into the recovery phase of KD (4). A previous untargeted metabolomics study profiling metabolic alterations in KD patients before and after intravenous immunoglobulin combined with aspirin treatment demonstrated that bile acid disorder represents the predominant metabolic perturbation in acute KD, with disease pathogenesis highly enriched in the bile acid biosynthesis pathway; bile acid metabolic dysregulation was significantly alleviated after treatment (5). The present study focuses on metabolic disturbances in primary bile acid metabolism and identifies TCA as a biomarker in the acute phase (9). Our results revealed that TCA was positively correlated with TBA and AST, indicating that hepatic inflammation was associated with TCA release. As an independent hepatic profibrotic factor, TCA upregulates collagen and actin expression, thereby exacerbating liver fibrosis. It also modulates the secretion of cytokines and chemokines, promotes hepatic neutrophil infiltration, and induces inflammatory liver injury. Clinically, elevated TCA levels have been observed in non-survivors of sepsis compared with survivors, with TCA levels positively correlated with bilirubin concentrations (16). According to the results of our study, TCA levels are positively correlated with proinflammatory cytokines (IL-6, IL-8) and inflammatory markers (Neu, CRP), while negatively correlated with the anti-inflammatory cytokine IL-10. We hypothesize that KD-associated hepatic vascular inflammatory injury may synergize with elevated TCA levels to amplify systemic inflammatory responses via vascular fibrotic remodeling, although this mechanistic hypothesis requires further validation in future investigations.

Amino acids function as fundamental building blocks of proteins and also exert critical effects on cardiovascular homeostasis. Research interest in amino acid profiles and their metabolic pathways continues to expand, as amino acids are not only regarded as candidate risk factors and biomarkers for atherosclerotic cardiovascular disease (17), but may also participate in immune regulation and inflammatory cascades to drive the initiation and progression of coronary artery disease (13). Amino acid metabolism may influence vascular inflammation through the following mechanisms. Disruptions in amino acid metabolism lead to immune dysregulation by affecting the function of T cells, B cells, and macrophages. Subsequent mitochondrial dysfunction and increased ROS production can exacerbate oxidative stress-induced vascular damage (18). Metabolic disturbances are associated with reduced NO synthesis and upregulated expression of adhesion molecules (13), influencing the transition of vascular smooth muscle cells from a contractile phenotype to a proinflammatory phenotype, and are linked to vascular endothelial dysfunction (19).

As key aromatic amino acids, phenylalanine, tyrosine and tryptophan participate in a wide range of physiological processes. Metabolic disorders of aromatic amino acids are often associated with oxidative stress, inflammatory injury and arterial lesions in the cardiovascular system (20). Serum levels of phenylalanine, tyrosine, and tryptophan constitute independent risk factors for traditional coronary artery disease, while tyrosine levels are negatively correlated with the Gensini score. Endogenous tyrosine metabolism has been shown to influence the activity of indoleamine 2,3-dioxygenase (IDO). Proinflammatory cytokine stimulation markedly upregulates IDO expression, leading to excessive tyrosine consumption and its conversion to quinolinic acid. This process aggravates inflammatory injury of the vascular bed and accelerates atherosclerotic progression (21). In mice models, IDO knockout leads to abnormal IL-10 expression and alleviates early atherosclerosis (22). Multiple studies focusing on immune disorders and cardiovascular diseases have demonstrated that tyrosine and its oxidation product 3-nitrotyrosine show significant correlations with proinflammatory cytokines (23–26). In our study, we identified that perturbed metabolites in acute-phase KD were enriched within the tyrosine metabolic pathway. Tyrosine levels were positively correlated with anti-inflammatory cytokines and negatively correlated with proinflammatory cytokines. These findings support the notion that tyrosine participates in immunosuppressive regulation and mitigates vascular inflammatory injury (27).

Arginine is a non-essential amino acid that participates in nitric oxide (NO) synthesis via endothelial NO synthase (eNOS) (13). NO relaxes vascular smooth muscle, dilates blood vessels, and inhibits leukocyte and platelet aggregation, thereby maintaining vascular homeostasis. Insufficient L-arginine levels or impaired NOS activity may affect NO bioavailability, and such alterations are associated with elevated vascular resistance (28). Histamine is the central metabolite in histidine metabolism; it has been implicated in inflammatory responses and may contribute to endothelial injury in concert with mast cells. Serum and synovial fluid histamine concentrations are elevated in patients with rheumatoid arthritis (RA), and *in vitro* co-culture experiments demonstrate increased IL-6 production within mast cells. In patients with hypertension, histamine levels are positively correlated with ESR. Combined with other immune markers, these indicators may facilitate evaluation of disease progression (29, 30). Histamine receptor blockers have been shown to exert protective effect against endothelial dysfunction in conditions such as myocardial ischemia, ischemia-reperfusion injury, and heart failure. Multiple studies have identified that arginine and histidine metabolic pathways are associated with coronary artery damage in children with KD by analyzing differential metabolites in peripheral blood (31). Levels of these metabolites decline during acute KD and recover following IVIG therapy; low abundance of histidine-related metabolites may be associated with refractory KD and the development of CAL. In addition, tryptophan metabolism primarily produces AhR ligands through the tryptophan ketone, serotonin, and indole-3-acetate pathways. AhR activation modulates B-cell polarization and promotes IL-10 secretion by B cells (32, 33). As an indispensable energy substrate for B-cell activation, glutamine supports B-cell survival and normal function by fuelling the tricarboxylic acid cycle, sustaining biomass synthesis, and maintaining redox homeostasis (34, 35). Glutamine deprivation or pharmacological/genetic inhibition of rate-limiting enzymes for glutamine catabolism leads to endothelial dysfunction. Glutamine levels are negatively correlated with inflammatory cytokines such as IL-6 and CRP, and glutamine may exert a protective effect under infectious conditions (36, 37). Clinical research indicate that glutamine can improve left ventricular systolic function following myocardial infarction, and there is a significant negative correlation between the glutamine-to-glutamate ratio and the phenotype of coronary artery disease (38, 39). Our exploratory findings reveal that multiple metabolic pathways are implicated in the pathogenesis of acute-phase KD. We further found that L-arginine and glutamine were significantly decreased and histamine was significantly increased during the acute phase. Histamine was positively correlated with proinflammatory cytokines, while glutamine was positively correlated with anti-inflammatory cytokines. We hypothesize that superantigen stimulation during acute KD initiates vascular endothelial injury and upregulates histidine decarboxylase activity in mast cells. Endothelial damage facilitates the accumulation of histamine and other metabolites, potentially increasing systemic vascular permeability. Meanwhile, these metabolites may synergize with proinflammatory cytokines to trigger diffuse endothelial dysfunction.

In summary, our study employed an untargeted metabolomics approach to profile plasma small-molecule metabolites in children with KD, revealing changes in the metabolic profile during the acute phase of a systemic inflammatory response. We acknowledge that the sample size of this study is relatively small, which inevitably raises concerns regarding overfitting during multivariate model construction. To rigorously address this issue, we employed two complementary cross-validation strategies. First, a 200-times repeated 7-fold cross-validation of the preselected six-metabolite logistic regression model yielded a bias-corrected mean AUC of 0.921, indicating that the apparent AUC (0.984) carried a modest degree of optimism. However, because the six candidate metabolites were initially selected from the full dataset using univariate ROC screening, this first cross-validation procedure did not fully account for the optimism introduced by the feature-selection step itself. To address this additional source of bias, we performed a more stringent nested cross-validation in which the univariate feature selection was repeated within each training fold of the outer cross-validation loop. The pooled AUC from this nested procedure was 0.889, which remains well above 0.5 and indicates that the six-metabolite panel retains strong discriminative ability even after full correction for both model fitting and feature-selection optimism. The true predictive performance of this model still holds promise in clinical settings. However, precise clinical application requires independent prospective validation in a larger external cohort. Owing to the reliance on HMDB database annotation, a small number of identified metabolites may originate from plants or non-human species, potentially causing false-positive results. A small number of compounds that are potentially exogenous or misannotated were not adopted for downstream biological interpretation. After untargeted metabolomic analysis, the samples were not further examined using targeted metabolomics or colorimetric detection, and large-scale targeted metabolomic data to validate the diagnostic value of each metabolite are lacking. Second, our study did not systematically address samples from patients with complications such as KD complicated by CAL or KD shock syndrome, nor did it describe post-treatment metabolite changes or the dynamic changes of metabolites at different stages of KD. Since the control group in our study consisted of healthy children, the findings can only illustrate the characteristic metabolic changes associated with acute systemic inflammation. Including children with infections caused by various pathogens as controls in future studies could strengthen the evidence for disease-specific metabolic features. Collectively, large-scale targeted metabolomic analyses of KD samples with more complications and across different stages will be needed to more fully reflect the value of these metabolic markers. In addition, external cohort validation is required to reduce feature selection bias, and the incorporation of external validation datasets will further improve the generalizability of our diagnostic models and study conclusions.

## 5 Conclusion

This exploratory study revealed characteristic perturbations of multiple metabolism-related pathways in the plasma of children with acute-phase KD. Six differential metabolites, including L-tyrosine, L-tryptophan, glutamine, histidine, histamine, and taurocholic acid, may serve as promising biomarkers for acute KD. Future studies are required to clarify the causal relationship between these core metabolites and KD pathogenesis, conduct large-scale targeted metabolomic validation, and systematically explore the potential molecular mechanisms by which these metabolites regulate vascular inflammatory injury in KD.

## Data Availability

The datasets presented in this study can be found in online repositories. The names of the repository/repositories and accession number(s) can be found below: The metabolomics data presented in our study are deposited in the Biological Project Library in National Genomics Data Center, China National Center for Bioinformation/Beijing Institute of Genomics, Chinese Academy of Sciences (Accession: PRJCA069273) that are publicly accessible at https://ngdc.cncb.ac.cn/bioproject.
