# Supplementary material for: Untargeted metabolomics reveals differential metabolic pathways and biomarkers in the acute phase of Kawasaki disease
Source: Front Med (Lausanne). 2026 Aug 26;13:1907194. doi: 10.3389/fmed.2026.1907194 (PMC13553804; doi:10.3389/fmed.2026.1907194)
Supplement: Supplementary file 1 [file Table1.docx]

| **TABLE S1** Nested cross‑validation of six‑metabolite panel | |
| --- | --- |
| Outer fold | AUC |
| 1 | 1.000 |
| 2 | 1.000 |
| 3 | 0.833 |
| 4 | 1.000 |
| 5 | 1.000 |
| 6 | 0.833 |
| 7 | 0.778 |
| 8 | 0.722 |
| 9 | 0.833 |
| 10 | 1.000 |
| Pooled (overall) AUC | 0.889 |
| Bootstrap 95% CI | 0.798 – 0.968 |
| Bootstrap CI was calculated with 2,000 resamples using the percentile method on the pooled test-set predictions across all outer folds. | |
